# Understanding the difference in symptoms and outcomes between glioblastoma patients diagnosed based on histological or molecular criteria: a retrospective cohort analysis from the Histo-Mol GBM collaborative

**DOI:** 10.1007/s11060-025-05364-8

**Published:** 2026-01-08

**Authors:** Stephen David Robinson, Sarah Kingdon, Sophie Therese Williams, Ciaran Scott Hill, Matthew Williams, Edward Chandy, Giles Critchley

**Affiliations:** 1 Sussex Cancer Centre, University Hospitals Sussex NHS Foundation Trust, Brighton, BN25BE UK; 2 Department of Biochemistry and Biomedicine, University of Sussex, Falmer, Brighton, UK; 3 Tessa Jowell Brain Cancer Mission, London, UK; 4 NHS Lothian, Edinburgh Cancer Centre, Edinburgh, UK; 5 NHS Greater Glasgow and Clyde, Beatson West of Scotland Cancer Centre, Glasgow, UK; 6 Division of Clinical Medicine, School of Medicine and Population Health, University of Sheffield, Sheffield, UK; 7 Sheffield Teaching Hospitals Foundation Trust, Weston Park Cancer Centre, Sheffield, UK; 8 National Hospital for Neurology and Neurosurgery, Brain Tumour Service (Neuro-Oncology), London, UK; 9 Department of Radiotherapy, Imperial College Healthcare NHS Trust, Charing Cross Hospital, London, UK; 10 Imperial College London, Computational Oncology Laboratory, Institute of Global Health Innovation, London, UK; 11 Centre for Medical Imaging Computing, University College London, London, UK; 12 Department of Neurosurgery, University Hospitals Sussex NHS Foundation Trust, Brighton, UK; 13 Section of Neurosurgery, Department of Surgical Sciences, Dunedin School of Medicine, University of Otago, Dunedin, New Zealand

**Keywords:** Glioblastoma, Molecular glioblastoma, Survival, Real-world evidence, Histology, Classification

## Abstract

**Purpose:**

Since the 2021 World Health Organisation (WHO) classification, glioblastoma could be diagnosed based on classical histological features (hGBM) or molecular criteria (mGBM). However, prior studies included patients who required reclassification as a mGBM, potentially biasing survival analyses. The Histo-Mol GBM collaborative performed an international multicentre retrospective real-world cohort study of glioblastoma patients diagnosed according to WHO CNS 5.

**Methods:**

We identified consecutive patients diagnosed in 2021 with IDH wildtype glioblastoma according to WHO CNS 5. Clinicopathological, treatment, and survival data were collected and compared between mGBM and hGBM.

**Results:**

1828 patients diagnosed with glioblastoma were included. 75 mGBM patients (8.4% of tested patients) were identified, with no difference in age (median 61 vs 64, *p* = 0.057), gender (*p* = 0.937), or proportion with performance status 0–1 (82.7% vs 68.3%, *p* = 0.052) compared to hGBM. mGBM patients had an extended interval from MRI to surgery (median 23 vs 14 days, *p* < 0.001) and more frequently underwent biopsy (69.3% vs 30.3%, *p* < 0.001), but equivalent proportions received oncological treatment (80.0% vs 78.7%, *p* = 0.784). Overall survival (OS) from surgery was not different (*p* = 0.063). However, OS from initial MRI, stratified by surgical extent, demonstrated improved OS for mGBM patients (hazard ratio (HR) 0.56, 95% confidence interval (CI): 0.43–0.73). Propensity score matching identified improved survival following resection (HR 0.48, 95% CI: 0.24–0.95; median OS: 26.0 versus 14.0 months, *p* = 0.031) but not biopsy (HR 1.10, 95% CI: 0.71–1.72).

**Conclusion:**

In this large real-world cohort, mGBMs had longer OS than hGBMs following resection with implications for prognostication and clinical decision making.

**Supplementary Information:**

The online version contains supplementary material available at 10.1007/s11060-025-05364-8.

## Introduction

Advances in understanding the molecular drivers of gliomagenesis has led to significant changes in brain tumour classification. Following the “Consortium to Inform Molecular and Practical Approaches to CNS Tumor Taxonomy” (cIMPACT-NOW) [1] and the 2021 World Health Organisation (WHO) Classification of Tumours of the Central Nervous System (CNS) [2], glioblastoma could be diagnosed by meeting histopathological criteria (necrosis and microvascular proliferation, hGBM) or identifying specific molecular features (mGBM).

These classifications [2, 3] define mGBM as an adult-type isocitrate dehydrogenase-wildtype (IDHwt) and histone H3 wildtype diffuse astrocytic glioma with molecular testing identifying at least one of: a telomerase reverse transcriptase promoter (pTERT) mutation, epidermal growth factor receptor (EGFR) amplification, or the combined gain of chromosome 7 and loss of chromosome 10 (Chr 7+/10-), and without meeting histopathological criteria. This classification change followed the identification that patients with histologically low grade IDHwt tumours with these molecular features had worse survival than would be expected for IDHwt tumours without these features [4–6].

However, prior studies included mGBM patients who were diagnosed over extended time periods, often spanning multiple diagnostic classification changes [7–12]. This required patient reclassification and often included historical control arms, which could have introduced bias into subsequent survival analyses comparing mGBM and hGBM patients [13].

To overcome previous limitations, we performed an international multicentre retrospective real-world cohort study, including consecutive IDHwt glioblastoma patients diagnosed in a single year according to WHO CNS 5, to compare presentation, tumour, and treatment characteristics plus survival outcomes between mGBM and hGBM patients.

## Materials and methods

We followed Strengthening Reporting of Observational Studies in Epidemiology (STROBE) [14] guidelines for this retrospective cohort study.

### Study approval

This study was approved by the University Hospitals Sussex (UHSussex) Clinical Outcomes and Effectiveness Committee (ref:1862) who confirmed exemption for gaining patient consent. Institutional review board approval, including information governance agreement, was required from each centre. New Zealand approval was provided from the Health and Disabilities Ethics Committee (ref:2024EXP21165) and the University of Otago Human Ethics Committee (Health) (ref:HD24/002). This study was conducted in accordance with the Declaration of Helsinki and all national and local regulations.

### Participants

We identified consecutive patients diagnosed with an IDHwt glioblastoma, according to WHO CNS 5, from 51 participating centres across three countries (Appendix 2), who underwent their diagnostic procedure between 01/01/2021–31/12/2021. Patients were identified and cross-referenced from local neuropathology, neurosurgery, and/or neuro-oncology records. Patients who had opted out of health record sharing through the United Kingdom National Data Opt-Out scheme (or equivalent) were excluded. Patients were diagnosed and treated according to local protocol.

### Data collection

We collected patient demographics, alongside presentation, tumour, and treatment characteristics, plus survival data (Appendix 3) locally from clinical records. Data was uploaded and managed using REDCap [15] electronic data capture tools, a secure online application designed for participant-based research, hosted at UHSussex.

Molecular testing was performed according to local practice. O6-methylguanine methyltransferase (MGMT) promoter methylation status was categorised as unmethylated (0–10%) or methylated ( > 10%) [16]. The surgical target was the contrast enhancing tumour if present. Given the challenges assessing extent of resection (EOR) retrospectively, especially for non-contrast enhancing tumours, surgery was classified as biopsy or resection (the therapeutic removal of any volume of tumour). Exploratory analysis using a modified RANO resect criteria [17] (partial resection < 80%, subtotal resection 80–94%, near total resection 95–99% gross total resection 100%) was also performed. Oncological treatment was categorised as: none, temozolomide alone, hypofractionated radiotherapy (40–53 Gray in > 2 Gray/fraction), hypofractionated chemoradiotherapy (hypofractionated radiotherapy plus concurrent temozolomide), conventionally fractionated chemoradiotherapy (54–60 Gray in 1.8–2 Gray/fraction plus concurrent temozolomide), or other treatment. Treatment was further categorised as: surgery only, intermediate (oncological treatment not meeting the criteria for aggressive), and aggressive (radiotherapy ≥40 Gray with concurrent temozolomide) [18].

Overall survival (OS) was calculated from date of surgery or date of MRI identifying a brain lesion subsequently confirmed to be glioblastoma (as specified) until death from any cause or censored at date of last follow up (neurosurgical/neurooncology clinic or brain MRI). Progression free survival (PFS) was calculated from date of surgery to MRI defined or neurosurgeon/neurooncologist defined clinical progression or censored at date of death/last follow up.

### Statistical analysis

Statistical analysis was performed using SPSS statistical software (version 29.0.2.0, Chicago, IL). Differences in categorical variables were assessed by chi-squared test, whilst continuous variables were assessed by Mann-Whitney U test. Standardised mean difference was calculated using Cohen’s d or Glass’s delta as appropriate. Length of follow up was calculated using the reverse Kaplan-Meier technique. Survival analysis was performed using Kaplan-Meier methodology with log rank test for significance. The Cox proportional hazards regression model was used for univariate and multivariate analysis (if univariate p-value < 0.05). Differences were considered statistically significant when the two-sided p-value was < 0.05. To detect a survival difference of 26% [13] between mGBM and hGBM patients at 90% power and an expected frequency of 10% mGBM, a total of 410 patients would be required at a p-value of 0.05 or 680 patients at a p-value of 0.005.

Propensity score matching (PSM) was performed to minimise the impact of confounding factors on survival analysis. Initially, a binomial logistic regression was performed using the identified patient, tumour, and treatment feature differences. Subsequently, PSM was performed based on the features identified from the binomial logistic regression plus those identified in the multivariate analysis and using a match tolerance of 0.05.

## Results

A total of 1828 patients were diagnosed with an IDHwt glioblastoma in 2021 from 51 centres (Appendix 1, 2). Rates of molecular testing ranged from 0 to 100% across centres (median 38% of patients tested) (Supplementary Figure 1) and 895 patients (49% of patients) underwent molecular testing during their diagnostic pathway. Of these, 75 patients were diagnosed with a mGBM (8.4%), with a similar proportion of mGBM patients (15/173, 8.7%) identified from the six centres who performed molecular testing in all patients.

### Comparison of patient and tumour characteristics of mGBM and hGBM patients

The majority of mGBM patients were male with no difference compared to hGBM (64% vs 64%, *p* = 0.937) and with a similar age at presentation (median 61 vs 64 years, *p* = 0.057). Additionally, there was no difference in WHO performance status (PS) at presentation (*p* = 0.152), with similar proportions of patients with PS 0–1 (83% vs 68%, *p* = 0.052) (Table 1).

**Table 1:** Comparison of patient, tumour, treatment, progression and follow up characteristics between molecular glioblastoma patients and histological glioblastoma patients with the significant features highlighted in bold

Characteristic	Unmatched				PSM matched			
	mGBM n (%)	hGBM n (%)	p-value	SMD	mGBM n (%)	hGBM n (%)	p-value	SMD
**Number of patients**	*N* = 75	*N* = 1753			*N* = 75	*N* = 67		
*Patient Factors*								
Gender:								
Male	48 (64.0%)	1114 (63.5%)	0.937	0.009	48 (64.0%)	44 (65.7%)	0.835	0.035
Female	27 (36.0%)	639 (36.5%)			27 (36.0%)	23 (34.3%)		
Age (years):								
Median	61	64	0.057	0.230	61	62	0.954	0.047
Range	24–87	18–88			24–87	24–83		
WHO PS at diagnosis:								
0	32 (42.7%)	498 (28.4%)			32 (42.7%)	12 (18.0%)		
1	30 (40.0%)	700 (39.9%)			30 (40.0%)	36 (53.7%)		
2	6 (8.0%)	244 (13.9%)	0.152	0.300	6 (8.0%)	11 (16.4%)	**0.007**	**0.581**
3	2 (2.7%)	63 (3.6%)			2 (2.7%)	5 (7.5%)		
4	0 (0.0%)	12 (0.7%)			0 (0.0%)	0 (0%)		
Unknown	5 (6.7%)	236 (13.5)			5 (6.7%)	3 (4.5%)		
WHO PS at diagnosis:								
0–1	62 (82.7%)	1198 (68.3%)	0.052	0.235	62 (82.7%)	48 (71.6%)	**0.041**	**0.424**
2+	8 (10.7%)	319 (18.2%)			8 (10.7%)	16 (23.9%)		
Unknown	5 (6.7%)	236 (13.5%)			5 (6.7%)	3 (4.5%)		
Presenting symptom - Seizure:								
Yes	31 (41.3%)	451 (25.7%)	**0.003**	0.357	31 (41.3%)	39 (58.2%)	**0.045**	**0.340**
No	44 (58.7%)	1302 (74.3%)			44 (58.7%)	28 (41.8%)		
Presenting symptom - Motor:								
Yes	16 (21.3%)	667 (38.0%)	**0.003**	0.344	16 (21.3%)	13 (19.4%)	0.776	0.048
No	59 (78.7%)	1086 (62.0%)			59 (78.7%)	54 (80.6%)		
Presenting symptom - Sensory:								
Yes	16 (21.3%)	194 (11.1%)	**0.006**	0.327	16 (21.3%)	13 (19.4%)	0.776	0.048
No	59 (78.7%)	1559 (88.9%)			59 (78.7%)	54 (80.6%)		
Presenting symptom - Sensory:								
Yes	16 (21.3%)	194 (11.1%)	**0.006**	0.327	16 (21.3%)	13 (19.4%)	0.776	0.048
No	59 (78.7%)	1559 (88.9%)			59 (78.7%)	54 (80.6%)		
Presenting symptom - Speech:								
Yes	11 (14.7%)	510 (29.1%)	**0.007**	0.318	11 (14.7%)	13 (19.4%)	0.452	0.126
No	64 (85.3%)	1243 (70.9%)			64 (85.3%)	54 (80.6%)		
Presenting symptom - Cognition:								
Yes	8 (10.7%)	475 (27.1%)	**0.002**	0.370	8 (10.7%)	17 (25.4%)	**0.022**	**0.473**
No	67 (89.3%)	1278 (72.9%)			67 (89.3%)	50 (74.6%)		
Presenting symptom - Behaviour:								
Yes	4 (5.3%)	134 (7.6%)	0.458	0.087	4 (5.3%)	4 (6.0%)	0.869	0.027
No	71 (94.7%)	1619 (92.4%)			71 (94.7%)	63 (94.0%)		
Presenting symptom - Visual:								
Yes	5 (6.7%)	168 (9.6%)	0.398	0.100	5 (6.7%)	8 (11.9%)	0.277	0.210
No	70 (93.3%)	1585 (90.4%)			70 (93.3%)	59 (88.1%)		
Presenting symptom - Headache:								
Yes	15 (20.0%)	600 (34.2%)	**0.011**	0.300	15 (20.0%)	25 (37.3%)	**0.022**	**0.430**
No	60 (80.0%)	1153 (65.8%)			60 (80.0%)	42 (62.7%)		
Presenting symptom – Reduced GCS:								
Yes	2 (2.7%)	75 (4.3%)	0.496	0.080	2 (2.7%)	1 (1.5%)	0.627	0.081
No	73 (97.3%)	1678 (95.7%)			73 (97.3%)	66 (98.5%)		
Presenting symptom - Incidental:								
Yes	4 (5.3%)	23 (1.3%)	**0.005**	0.178	4 (5.3%)	0 (0%)	0.055	0.236
No	71 (94.7%)	1730 (98.7%)			71 (94.7%)	67 (100%)		
Presenting symptom – Other:								
Yes	15 (20.0%)	236 (13.5%)	0.107	0.190	15 (20.0%)	12 (17.9%)	0.751	0.053
No	60 (80.0%)	1517 (86.5%)			60 (80.0%)	55 (82.1%)		
Corticosteroids at diagnosis:								
Yes	43 (57.3%)	1392 (79.4%)	** < 0.001**	0.571	43 (57.3%)	56 (83.6%)	**0.001**	**0.498**
No	30 (40.0%)	322 (18.4%)			30 (40.0%)	11 (17.9%)		
Unknown	2 (2.7%)	39 (2.2%)			2 (2.7%)	0 (0%)		
Anti-epileptic drugs at diagnosis:								
Yes	45 (60.0%)	780 (44.5%)	**0.007**	0.321	45 (60.0%)	55 (82.1%)	**0.005**	**0.433**
No	29 (38.7%)	960 (54.8%)			29 (38.7%)	12 (17.9%)		
Unknown	1 (1.3%)	13 (0.7%)			1 (1.3%)	0 (0%)		
*Tumour Factors*								
Tumour Location:								
Left	39 (52.0%)	788 (45.0%)			39 (52.0%)	35 (52.2%)		
Right	31 (41.3%)	868 (49.5%)	0.373	0.097	31 (41.3%)	29 (43.3%)	0.847	0.040
Midline/bilateral	5 (6.7%)	95 (5.4%)			5 (6.7%)	3 (4.5%)		
Tumour Location*:								
Frontal	25 (33.3%)	653 (37.3%)	0.492	0.081	25 (33.3%)	22 (32.8%)	0.950	0.010
Parietal	33 (44.0%)	530 (30.3%)	**0.012**	**0.298**	33 (44.0%)	27 (40.3%)	0.656	0.074
Temporal	37 (49.3%)	754 (43.0%)	0.279	0.128	37 (49.3%)	30 (44.8%)	0.587	0.091
Occipital	5 (6.7%)	157 (9.0%)	0.494	0.081	5 (6.7%)	10 (14.9%)	0.110	0.329
Cerebellum	3 (4.0%)	22 (1.3%)	**0.045**	**0.247**	3 (4.0%)	0 (0%)	0.098	0.203
Brainstem	4 (5.3%)	42 (2.4%)	0.112	0.192	4 (5.3%)	3 (4.5%)	0.814	0.039
Multifocal/multicentric^^^ tumour:								
Yes	28 (37.3%)	423 (24.1%)	**0.010**	**0.308**	28 (37.3%)	19 (28.4%)	0.257	0.184
No	47 (62.7%)	1328 (75.8%)			47 (62.7%)	48 (71.6%)		
Unknown	0 (0.0%)	2 (0.1%)			0 (0.0%)	0 (0%)		
Contrast enhancing on MRI:								
Yes	54 (72.0%)	1667 (95.1%)	** < 0.001**	**1.236**	54 (72.0%)	66 (98.5%)	** < 0.001**	**0.571**
No	20 (26.7%)	64 (3.7%)			20 (26.7%)	1 (1.5%)		
Unknown	1 (1.3%)	22 (1.3%)			1 (1.3%)	0 (0%)		
Histological Grade:								
2	13	0 (0.0%)			13	0 (0.0%)		
3	62	31 (1.8%)	** < 0.001**	**5.659**	62	0 (0.0%)	** < 0.001**	**2.992**
4	0 (0.0%)	1688 (96.3%)			0 (0.0%)	67 (100%)		
Unknown/Ungraded	0 (0.0%)	34 (1.9%)			0 (0.0%)	0 (0%)		
Molecular markers – pTERT^#^:								
Yes	59 (89.4%)	507 (78.4%)	**0.035**	**1.107**	59 (89.4%)	52 (91.2%)	0.828	0.056
No	7 (10.6%)	140 (21.6%)			7 (10.6%)	5 (8.8%)		
Not tested	9	1075			9	10		
Molecular markers – EGFR^#^:								
Yes	28 (42.4%)	240 (37.3%)			28 (42.4%)	25 (41.0%)		
No	38 (67.9%)	403 (62.7%)	0.416	1.031	38 (67.9%)	36 (59.0%)	0.922	0.021
Not tested	9	1060			9	4		
Molecular markers – Chr 7+/10-^#^:								
Yes	19 (52.8%)	87 (36.9%)			19 (52.8%)	7 (70.0%)		
No	17 (47.2%)	149 (63.1%)	0.068	1.107	17 (47.2%)	3 (30.0%)	** < 0.001**	**0.586**
Not tested	39	1453			39	56		
MGMT promoter methylation:								
Unmethylated (0–10%)	62 (82.7%)	1241 (70.8%)			62 (82.7%)	60 (89.6%)		
Methylated ( > 10%)	13 (17.3%)	454 (25.9%)	0.096	0.225	13 (17.3%)	7 (10.4%)	0.239	0.181
Unknown	0 (0.0%)	58 (3.3%)			0 (0.0%)	0 (0%)		
*Treatment Factors*								
Time from MRI to surgery:								
Median (days)	23	14	** < 0.001**	**0.686**	23	17	**0.009**	**0.001**
Range (days)	0–869	0–1395			0–869	0–1395		
Extent of surgery:								
Biopsy	52 (69.3%)	532 (30.3%)			52 (69.3%)	39 (58.2%)		
Resection	23 (30.7%)	1214 (69.3%)	** < 0.001**	**0.847**	23 (30.7%)	28 (41.8%)	0.168	0.240
Unknown	0 (0.0%)	7 (0.4%)			0 (0.0%)	0 (0%)		
Time from surgery to radiotherapy:								
Median (days)	46	40	**0.002**	**0.381**	46	39	**0.004**	**0.406**
Range (days)	12–108	9–245			12–108	22–115		
Oncological treatment:								
None	15 (20.0%)	374 (21.3%)			15 (20.0%)	14 (20.9%)		
Temozolomide	4 (5.3%)	49 (2.8%)	0.784	0.015	4 (5.3%)	6 (9.0%)	0.920	0.017
Hypofractionated RT	8 (10.7%)	196 (11.2%)			8 (10.7%)	8 (11.9%)		
Hypofractionated CRT	10 (13.3%)	225 (12.8%)			10 (13.3%)	7 (10.4%)		
Conventional CRT	31 (41.3%)	752 (42.9%)			31 (41.3%)	31 (46.3%)		
Other treatments	7 (9.3%)	157 (9.0%)			7 (9.3%)	1 (3.3%)		
Oncological treatment:								
*Surgery only*	*15 (20.0%)*	*374 (21.3%)*			*15 (20.0%)*	*14 (20.9%)*		
*Intermediate*	*19 (25.3%)*	*402 (22.9%)*	*0.880*	*0.003*	*19 (25.3%)*	*15 (22.4%)*	*0.919*	*0.014*
*Aggressive*	*41 (54.7%)*	*977 (55.7%)*			*41 (54.7%)*	*38 (56.7%)*		
*Progression factors*								
Confirmed progression:								
Yes	57 (76.0%)	1294 (75.5%)	0.921	0.012	57 (76.0%)	50 (76.9%)	0.898	0.022
No	18 (24.0%)	420 (24.5%)			18 (24.0%)	15 (23.1%)		
Received 2^nd^ line treatment:								
Yes	28 (49.1%)	546 (42.2%)	0.300	0.140	28 (49.1%)	27 (54.0%)	0.615	0.097
No	29 (50.9%)	748 (57.8%)			29 (50.9%)	23 (46.0%)		
*Survival/Follow up*								
Patient surviving:								
Yes	11 (14.7%)	225 (12.8%)	0.643	0.055	11 (14.7%)	10 (14.9%)	0.965	0.007
No	64 (85.3%)	1528 (87.2%)			64 (85.3%)	57 (85.1%)		
Length of follow up:								
Median	33.8 months	34.2 months	0.584		33.8 months	30.9 months	0.262	
95% confidence interval	29.7–37.8	33.1–35.2			29.7–37.8	23.5–38.2		

* combined percentages may exceed 100 from tumours occupying multiple lobes. ^^^ Multifocal/multicentric tumours defined retrospectively based on the presence of multiple tumour foci (contiguous or non-contiguous) on magnetic resonance imaging. ^#^ percentages and statistics calculated for tested patients only. PSM: propensity score matched; mGBM: molecular glioblastoma; hGBM, histological glioblastoma; SMD: standardised mean difference; WHO PS: World Health Organisation performance status; GCS: Glasgow Coma Score; MRI: magnetic resonance imaging; pTERT: telomerase reverse transcriptase promoter mutation; EGFR: epidermal growth factor receptor amplification; Chr 7+/10-: combined gain of chromosome 7 and loss of chromosome 10; MGMT: O6-methylguanine methyltransferase; RT: radiotherapy alone; CRT: concurrent chemoradiotherapy; Other treatments: other oncological treatments (conventionally fractionated radiotherapy alone, palliative radiotherapy, etc

However, symptoms present at diagnosis differed, with more mGBM patients reporting seizures (41% vs 26%, *p* = 0.003), including an increase in pre-operative anti-epileptic drug (AED) prescriptions (60% vs 45%, *p* = 0.007), and sensory symptoms (21% vs 11%, *p* = 0.006), alongside more incidental diagnoses (5.3% vs 1.3%, *p* = 0.006). Further, fewer mGBM patients reported motor (21% vs 38%, *p* = 0.003), speech (15% vs 29%, *p* = 0.007), cognitive (11% vs 27%, *p* = 0.002), and headache (20% vs 34%, *p* = 0.011) symptoms at diagnosis, and had fewer pre-operative corticosteroids prescribed (57% vs 79%, *p* < 0.001) (Table 1).

Tumour location differed with greater numbers of mGBM tumours involving the parietal lobe (44% vs 30%, *p* = 0.012) and cerebellum (4.0% vs 1.3%, *p* = 0.045). Additionally, there were more multifocal/multicentric (37% vs 24%, *p* = 0.010) and non-contrast enhancing tumours (27% vs 3.7%, *p* < 0.001) in mGBM patients. Most mGBM patients’ tumours displayed histological features equating to grade 3 (62/75, 83%) with a minority of grade 2 tumours (13/75, 17%) (Table 1).

For the patients whose tumours underwent molecular testing, there was no difference in the proportion of tumours with methylated MGMT (17% vs 26%, *p* = 0.096), EGFR amplification (42% vs 37%, *p* = 0.416), or Chr 7+/10- (53% vs 37%, *p* = 0.068). However, more mGBM patients had tumours with a pTERT mutation (89% vs 78%, *p* = 0.035) (Table 1).

**Fig. 1:**
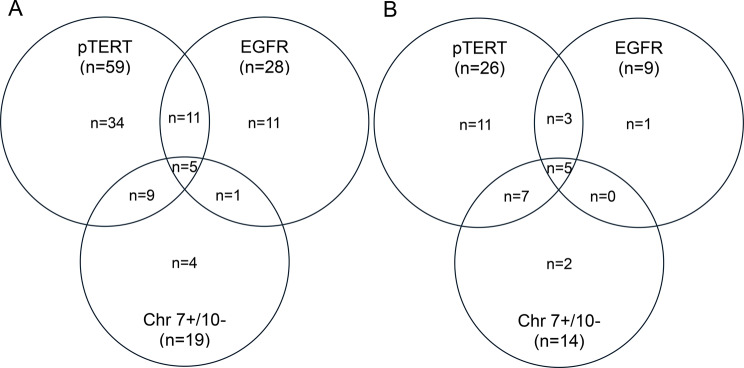
Venn diagrams highlighting the overlap in the identification of the defining molecular alterations for the molecular glioblastoma patients within **A**) all patients and **B**) the subgroup of patients with comprehensive molecular testing. pTERT: telomerase reverse transcriptase promoter mutation; EGFR: epidermal growth factor receptor amplification; chr 7+/10-: combined gain of chromosome 7 and loss of chromosome 10

Focussing on the mGBM cohort, Figure 1 demonstrates the composition of defining molecular alterations for the patients diagnosed with a mGBM. Of the 75 mGBM patients identified, 49 patients (65%) had a singular defining molecular feature identified, 21 (28%) had two features, and five (6.7%) had all three features (Fig. 1A). However, most patients’ tumours underwent partial molecular testing, with only 29 patients’ tumours (39%) undergoing comprehensive molecular characterisation. In this subgroup, 14 patients (48%) had a singular molecular feature, predominantly pTERT mutation only (11 patients, 38%), 10 patients (35%) had two features, and five patients (17%) had all three features identified.

### Comparison of treatment characteristics of mGBM and hGBM patients

Patients with mGBM had an extended interval from MRI to surgery (23 vs 14 days, *p* < 0.001). Additionally, the majority of mGBM patients had a biopsy compared to a much lower proportion of hGBM patients (69% vs 30%, *p* < 0.001) (Table 1). However, EOR was similar for patients receiving resection, even within the subgroup of contrast enhancing tumours (Supplementary Table 1). There was also an extended time from surgery to radiotherapy (if received) for mGBM patients (46 vs 40 days, *p* = 0.002) in keeping with a delay in receiving the final molecular histopathology report prior to definitive decision making. However, there was no difference in the oncological treatment received, with equal proportions of patients receiving any treatment (80% vs 79%, *p* = 0.784), and equivalent proportions receiving conventionally fractionated chemoradiotherapy (41% vs 43%, *p* = 0.784) (Table 1).

### Comparison of PFS and OS for mGBM and hGBM patients

**Fig. 2:**
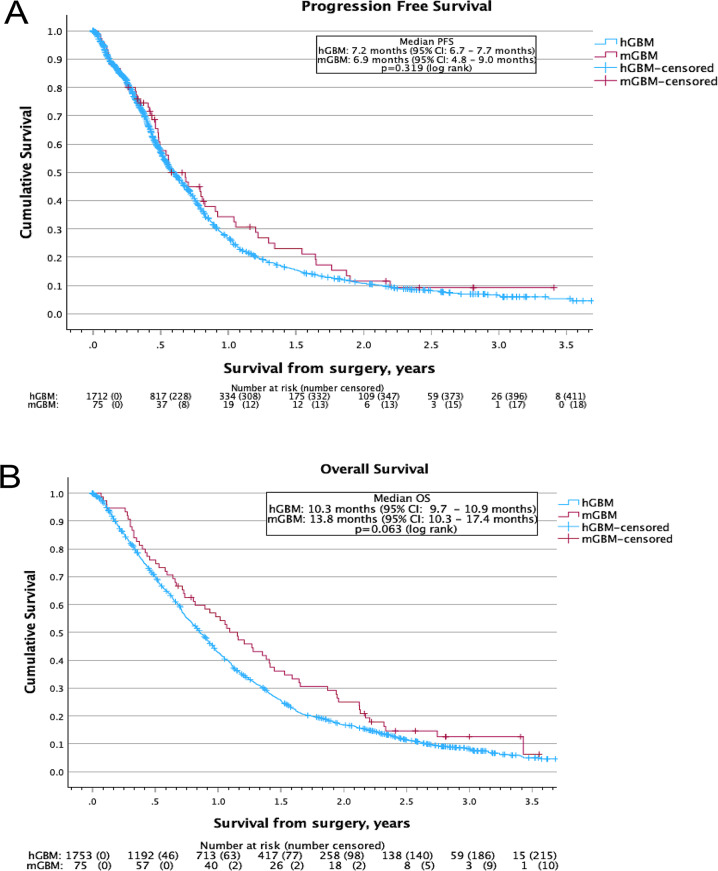

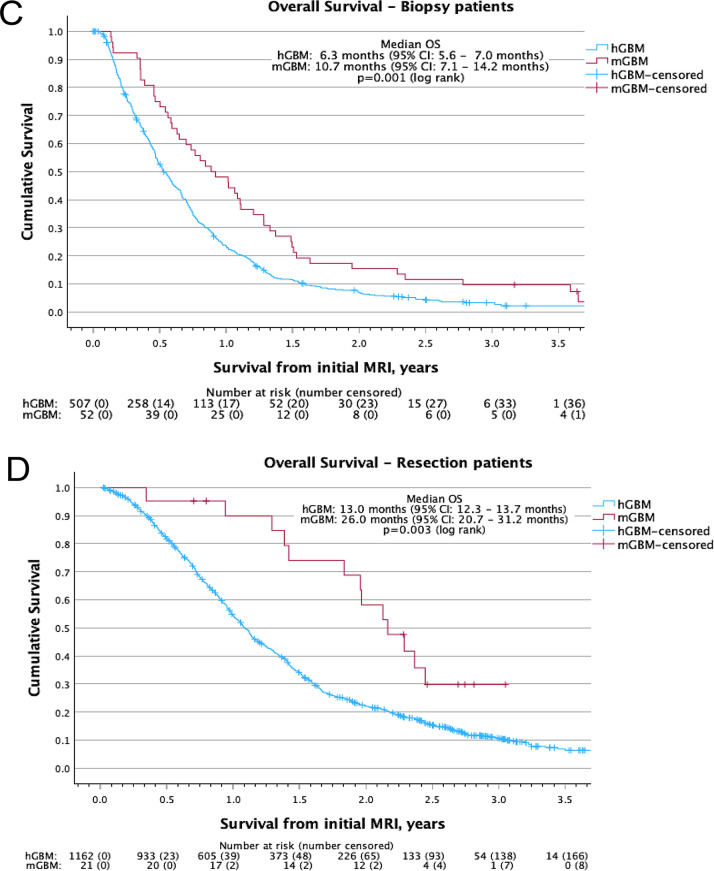
Kaplan-meier survival curves comparing survival between molecular glioblastoma patients and histological glioblastoma patients. **A**) progression free survival and **B**) overall survival from date of surgery. Overall survival from date of initial magnetic resonance imaging for **C**) patients who underwent biopsy and **D**) patients who underwent resection. PFS: progression free survival from date of surgery; hGBM: histological glioblastoma; mGBM: molecular glioblastoma; 95% ci: 95% confidence interval; os: overall survival; MRI: magnetic resonance imaging

Progressive disease (either clinical or radiological) was documented in around three quarters of mGBM and hGBM patients (76% vs 76%, *p* = 0.921) (Table 2) with similar median PFS from surgery of 6.9 months (95% confidence interval (CI) 4.8–9.0 months) for mGBM compared to 7.2 months (95% CI 6.7–7.7 months, *p* = 0.319) for hGBM patients (Fig. 2A). Following progression, similar proportions of patients received subsequent treatment (49% vs 42%, *p* = 0.300).

**Table 2:** Univariate and multivariate cox regression analysis for overall survival in molecular glioblastoma patients

Variable	Univariate		Multivariate	
	p value	Hazard ratio (95% CI)	p value	Hazard ratio (95% CI)
**Age:** **Continuous**	**0.007**	**1.03** **(1.01–1.06)**	0.462	
Gender: Male vs Female	0.968			
**Presenting symptom – Seizures:** **No vs Yes**	** < 0.001**	**0.36** **(0.20–0.63)**	0.474	
**Presenting symptom – Motor:** **No vs Yes**	**0.004**	**2.39** **(1.32–4.31)**	**0.018**	**2.71** **(1.19–6.16)**
Presenting symptom – Speech: No vs Yes	0.734			
Presenting symptom – Cognition: No vs Yes	0.152			
**Presenting symptom – Behaviour:** **No vs Yes**	** < 0.001**	**14.02** **(4.02–48.84)**	0.101	
**Presenting symptom – Vision:** **No vs Yes**	**0.046**	**2.61** **(1.02–6.68)**	0.405	
Presenting symptom – Headache: No vs Yes	0.052			
Presenting symptom – Sensory: No vs Yes	0.742			
**Presenting symptom – Reduced GCS:** **No vs Yes**	**0.021**	**5.57** **(1.29–23.99)**	**0.007**	**13.40** **(2.05–87.41)**
Presenting symptom – Incidental: No vs Yes	0.860			
Presenting symptom – Other: No vs Yes	0.451			
Corticosteroids at diagnosis: No vs Yes	0.334			
**AEDs at diagnosis:** **No vs Yes**	**0.002**	**0.44** **(0.26–0.74)**	**0.013**	**0.33** **(0.41–0.80)**
**WHO Performance status:** **0–1 vs 2+**	**0.006**	**3.27** **(1.46–7.56)**	0.059	
Site: Left vs Right vs Midline/Bilateral	0.139			
Frontal lobe: No vs Yes	0.545			
Parietal lobe: No vs Yes	0.806			
Temporal lobe: No vs Yes	0.431			
Occipital lobe: No vs Yes	0.283			
Cerebellum: No vs Yes	0.094			
Brainstem: No vs Yes	0.368			
**Multifocal/multicentric:** **No vs Yes**	**0.048**	**1.69** **(1.01–2.83)**	0.271	
Contrast enhancing on MRI: No vs Yes	0.125			
Grade: 2 vs 3	0.161			
MGMT methylation: No vs Yes	0.473			
**Surgery:** **Biopsy vs Resection**	**0.001**	**0.36** **(0.19–0.67)**	0.266	
**Oncological treatment:** **None vs Intermediate vs Aggressive**	** < 0.001**	**0.33** **(0.22–0.48)**	**0.010**	**0.46** **(0.25–0.83)**

Variables with p-value < 0.05 on univariate analysis were chosen for assessment in the multivariate analysis with the significant features highlighted in **bold**

95% CI: 95% confidence interval; GCS: Glasgow Coma Score; AEDs: Anti-epileptic drugs; WHO: World Health Organisation; MRI: Magnetic Resonance Imaging; MGMT: O6-methylguanine methyltransferase

At the time of analysis, equivalent proportions of patients remained alive (15% vs 13%, *p* = 0.643) with equal median length of follow up (33.8 vs 34.2 months, *p* = 0.584) (Table 1). Kaplan-Meier survival analysis demonstrated no clear difference between groups, with median OS from surgery of 13.8 months (95% CI: 10.3–17.4 months) for mGBM patients compared to 10.3 months (95% CI: 9.7–10.9 months, *p* = 0.063) for hGBM patients (Fig. 2B).

However, as demonstrated in Table 1, there was an increase in the time from MRI to surgery and differences in the surgery performed, both of which are likely to impact OS. Repeating the Kaplan-Meier analysis when computing OS from MRI scan and stratifying by surgical approach demonstrated that mGBM patients have improved OS compared to hGBM patients (hazard ratio (HR) 0.56, 95% CI: 0.434–0.730) (Fig. 2C,D). For patients who underwent neurosurgical biopsy (HR 0.61, 95% CI: 0.45–0.82), median OS from MRI for mGBM patients was 10.7 months (95% CI: 7.1–14.2 months) compared to 6.3 months (95% CI: 5.6 vs 7.0 months, *p* = 0.001) for hGBM patients (Fig. 2C). Whilst for patients who underwent resection (HR 0.45, 95% CI: 0.26–0.77), median OS from MRI was 26.0 months (95% CI: 20.7–31.2 months) for mGBM compared to 13.0 months (95% CI: 12.3–13.7 months, *p* = 0.003) for hGBM patients (Fig. 2D).

### Analysis of factors associated with OS in mGBM patients

Univariate Cox regression analysis identified that increasing age (*p* = 0.007), presenting with motor (*p* = 0.004), behavioural (*p* < 0.001), or visual symptoms (0.046), reduced Glasgow Coma Score (GCS) (*p* = 0.021), PS ≥ 2 (*p* = 0.006) or a multifocal/multicentric tumour (*p* = 0.048) were associated with worse OS for mGBM patients, whilst presenting with seizures (*p* < 0.001), receiving an anti-epileptic drug (AED) at diagnosis (*p* = 0.002), undergoing resection (*p* = 0.001), or increasing intensity of oncological treatment (*p* < 0.001) were associated with improved OS (Table 2).

On multivariate analysis, presenting with motor symptoms (HR 2.71, 95% CI: 1.19–6.16, *p* = 0.018) or reduced GCS (HR 13.40, 95% CI: 2.05–87.41, *p* = 0.007), receiving an AED at diagnosis (HR 0.33, 95% CI: 0.41–0.80, *p* = 0.013), and increasing intensity of oncological treatment (Aggressive > Intermediate > Surgery only; HR 0.46, 95% CI: 0.25–0.83, *p* = 0.010) remained as prognostic factors for mGBM patients (Table 2).

### Comparison of survival in propensity score matched mGBM and hGBM patients

To identify the features most associated with an mGBM diagnosis, a binomial logistic regression (Supplementary Table 2) was performed using the differences identified between mGBM and hGBM (Table 1). Subsequently, these features, plus the features associated with mGBM patient OS on multivariate analysis (Table 2), were used to perform PSM.

**Fig. 3:**
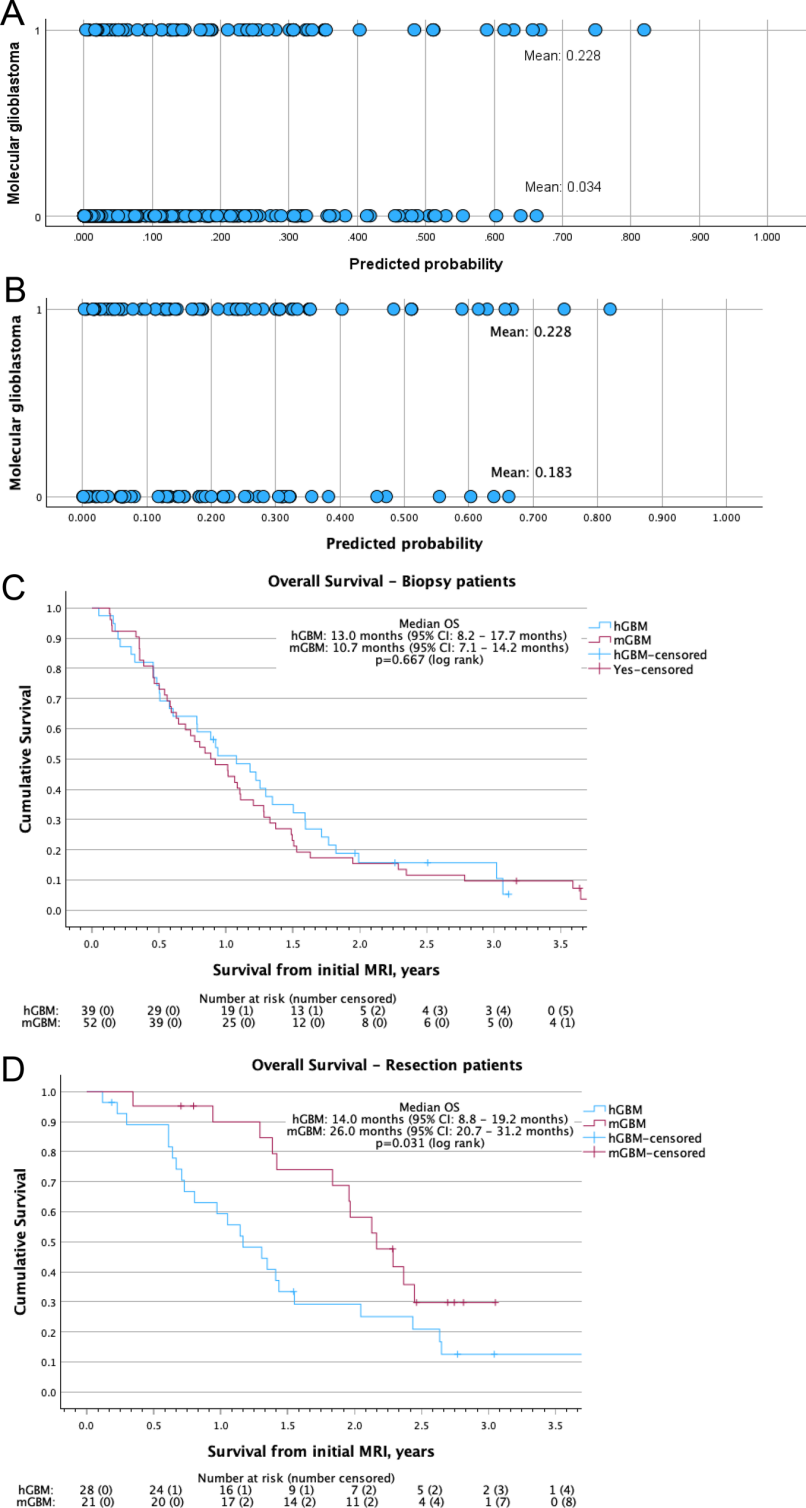
Propensity score matching confirms the overall survival differences between molecular glioblastoma patients and histological glioblastoma patients. Scatter plot comparing the distribution of predicted probabilities of being a molecular glioblastoma generated from the binomial logistic regression analysis for **A**) the whole cohort and **B**) the propensity score matched cohort. Kaplan-meier survival curves comparing the overall survival from date of initial magnetic resonance imaging between molecular glioblastoma patients and histological glioblastoma patients for **C**) patients who underwent biopsy and **D**) patients who underwent resection. 1 = molecular glioblastoma; 0 = histological glioblastoma; os: overall survival from date of surgery; hGBM: histological glioblastoma; mGBM: molecular glioblastoma; 95% ci: 95% confidence interval; MRI: magnetic resonance imaging

After applying PSM, the balance of covariates between groups improved and potential confounding was reduced (Fig. 3A,B). The improved similarities of the patient, tumour and treatment variables for the 75 mGBM patients and the 67 matched hGBM patients (Table 1) confirm the effectiveness of the matching, although some differences remained.

Comparing OS between these matched groups (Fig. 3C,D) identified no difference in survival for patients who undergo biopsy (HR 1.10, 95% CI: 0.71–1.72) with a median OS of 10.7 months (95% CI: 7.1–14.2 months) for mGBM patients compared to 13.0 months (95% CI: 8.2–17.7 months, *p* = 0.667) for hGBM patients (Fig. 3C). However, in patients who undergo resection, mGBM patients have a better prognosis (HR 0.48, 95% CI: 0.24–0.95), with a median OS of 26.0 months (95% CI: 20.7–31.2 months) compared to 14.0 months (95% CI: 8.8–19.2 months, *p* = 0.031) for hGBM patients (Fig. 3D).

## Discussion

The 2021 WHO CNS classification [2] marked a foundational shift in neuro-oncology, through including molecular features within a final integrated diagnosis. However, several studies have published discordant results when assessing whether mGBM patient survival is equivalent to hGBM patients [7, 10–13, 19–21]. One challenge of using retroactively reclassified patients, is that long study periods or comparisons with historical controls will potentially introduce bias due to temporal shifts in clinical management [22].

To overcome this challenge, we developed a large, international, multicentre cohort database comprising 1828 patients consecutively diagnosed within a calendar year with a pathologically confirmed IDHwt glioblastoma according to WHO CNS 5. This unique dataset enables population level comparisons of contemporaneous mGBM and hGBM patients, diagnosed and treated according to the same local protocols, using granular individual patient data.

Compared to previous studies [12, 23], we identified no difference in age, or other demographics, between mGBM and hGBM patients. However, we identified differences in clinical presentation, with mGBM patients more commonly describing seizures and sensory changes, but less frequently describing motor, speech or cognitive symptoms, similar to the study by Guo et al. [10], which likely explains the differences identified in AED and steroid prescriptions at diagnosis. This altered clinical presentation has implications for the diagnostic pathway as this symptom profile is less common with hGBM [24]. Furthermore, the lower prevalence of contrast enhancing lesions (72% vs 95%), increases the diagnostic uncertainty due to the overlap between these findings and encephalitis and/or cerebral vasculitis [25, 26]. This may explain the extended interval between MRI and surgery (median 9 days delay) for mGBM patients underscoring the need for robust diagnostic pathways to pathologically confirm radiologically “low-grade” appearances.

Consistent with published studies, we identified that mGBM patients were more likely to have multifocal/multicentric tumours (37% vs 24%) and undergo biopsy rather than resection (69% vs 30%) [11–13], but with similar EOR if resection was performed. These features, coupled with the previously described gliomatosis cerebri growth pattern [7, 12], has raised the concern that a subset of mGBM diagnoses may represent “under-sampled” hGBM [20]. Currently, standard surgical practice is to sample an area of contrast enhancement (where present), supplemented by further imaging modalities depending on local practice, that is presumed representative of the highest grade of the tumour [27]. However, whilst our initial analysis suggested improved outcomes for mGBM patients following biopsy (HR 0.61, 95% CI: 0.45–0.82), subsequent PSM demonstrated equivalent survival (HR 1.10, 95% CI: 0.71–1.72). This suggests that PSM removed unidentified confounders and highlights the difficulty in accurately identifying mGBM on biopsy samples.

The main finding from this study is that patients with mGBM have a longer OS compared to hGBM patients following resection (HR 0.45, 95% CI: 0.26–0.77), despite the similar EOR, a result confirmed by PSM (HR 0.48, 95% CI: 0.24–0.95). The substantial OS benefit (median OS 26.0 vs 13.0 months) following resection, supports the consideration of subsequent resection when technically feasible following an mGBM diagnosis on biopsy, to confirm the diagnosis and representing a form of molecularly-based decision-making [28].

This OS improvement aligns with a previous meta-analysis from 2023 [13], which reported improved survival on multivariate analysis (HR 0.61, 95% CI: 0.50–0.74), although most of the included studies identified no clear OS difference [7, 9, 10, 19, 23, 29, 30]. This discrepancy may be attributable to insufficient statistical power in earlier studies or variable proportions of patients undergoing resection across cohorts. Improved survival has also been noted in patients without contrast enhancement on MRI [31]. However, in this cohort, the majority of mGBM patients who underwent resection had contrast enhancing tumours (91%) suggesting that this variable does not account for the observed survival benefit. Additionally, while several studies reported less intensive oncological treatment for mGBM patients [7, 23, 29, 30], we identified differences in the diagnostic pathway that independently impact OS, potentially explaining some of the heterogeneity in prior results. Although the extended MRI interval in mGBM patients to surgery raises of lead time bias, the median difference of 9 days is unlikely to be the primary determinant of the survival difference.

Given the OS difference identified, we suggest routinely collecting information on molecular vs histological diagnosis of glioblastoma for trial participants and considering stratifying for mGBM vs hGBM at trial entry, given the risk that potentially important survival improvements may be obscured by imbalances in patients between treatment arms.

## Limitations

A limitation of this study stems from the cohort’s recruitment during 2021, a period during which many participating centres were still subject to operational pressures related to the SARS-COV2 pandemic [32, 33]. Notable, some centres reported reduced surgical capacity leading to a 50% reduction in the number of pathologically confirmed glioblastoma patients (personal communication). The potential exclusion of patients with poor prognostic characteristics may have favourably biased OS estimates. However, the reported PFS and OS for hGBM patients align with previously published results [18], suggesting that SARS-COV2 related adjustments had limited influence on survival in this cohort.

Further, WHO CNS 5 was published in June 2021 [2], although cIMPACT-NOW update 3 was published in 2018 [3]. Therefore, most centres were not performing routine molecular testing for all (*n* = 36 centres) or any (*n* = 9 centres) glioblastoma patients, which may have led to an under-diagnosis of mGBM in this and introduced selection bias regarding which patients underwent advanced testing. However, similar proportions of mGBM patients were identified at centres with complete molecular testing (8.7%) and the overall cohort (8.4%) providing confidence in this estimate. However, we advocate for comprehensive molecular testing as ~10–21% of mGBM patients would be missed by only assessing pTERT.

Finally, the small numbers of mGBM patients compared to hGBM limits the power of subgroup analyses, potentially obscuring true differences. This is especially challenging given the heterogeneity of mGBM, which includes six different combinations of EGFR amplification/pTERT/Chr 7+/10-, and the emerging evidence to suggest that pTERT only mGBM may have different biology from other mGBM patients [34].

## Conclusion

In conclusion, we present a comparison of mGBM and hGBM patients diagnosed according to WHO CNS 5 using a large real-world cohort. We demonstrate that glioblastoma patients diagnosed according to molecular features, are uncommon ( < 10%), and differ compared to those diagnosed based on classical histological criteria. We also identify that mGBM patients had longer OS than hGBM patients following resection with implications for clinical decision making, prognostic estimates given to patients, and clinical trial enrolment.

## Electronic supplementary material

Below is the link to the electronic supplementary material.

Supplementary Material 1

Supplementary Material 2

Supplementary Material 3

Supplementary Material 4

Supplementary Material 5

## Data Availability

The summary data generated during the current study are available from the corresponding author on reasonable request. The complete data are not available due to patient confidentiality.
