# Supplementary material for: Understanding the difference in symptoms and outcomes between glioblastoma patients diagnosed based on histological or molecular criteria: a retrospective cohort analysis from the Histo-Mol GBM collaborative
Source: J Neurooncol. 2026 Jan 8;176(2):157. doi: 10.1007/s11060-025-05364-8 (PMC12783167; doi:10.1007/s11060-025-05364-8)
Supplement: Supplementary file 1 — Supplementary Material 1 [file 11060_2025_5364_MOESM1_ESM.docx]

**Title: Understanding the difference in symptoms and outcomes between glioblastoma patients diagnosed based on histological or molecular criteria: a retrospective cohort analysis from the Histo-Mol GBM collaborative.**

**Appendix 1. Histo-Mol GBM collaborative centres ordered by number of patients included:**

The Beatson West of Scotland Cancer Centre: Attika Chaudhary, Sean Farrell, Simon Lammy.

University Hospitals Birmingham NHS Foundation Trust: Reshma David, Neha Rathod, Vinton Cheng.

Newcastle upon Tyne Hospitals NHS Foundation Trust: Alex Byers, Noah Shi Jie Lee, Ian Coulter.

Leeds Teaching Hospitals NHS Trust: Michael Clark, Christopher Hooper, Kavi Fatania, Stuart Currie.

Auckland City Hospital: Jayne Sheridan, Malinda Lucas, Davina McAllister, Peter Heppner.

Clatterbridge Cancer Centre: Jillian Sokratous, Marcus Rathbone, Shaveta Mehta.

Kent Oncology Centre: Katherine Ryan, Louisa Black, Samantha Forner.

Nottingham University Hospitals NHS Trust: Ia Robert-Montaner, Hawawu Muazu, Paul McDonnell, Daniele Scotto, Mihir Kocherlakota, Jake Hewitt, Joon Ho, Sangary Kathirgamakarthigeyan.

Cambridge University Hospitals NHS Foundation Trust: Emily Lachmann, Rebecca Sen, Mareike Thompson.

National Hospital for Neurology and Neurosurgery: Adrianna Wong, Henry Ajah, Bernadette Kovacs, Ciaran Hill.

Weston Park Cancer Hospital: Lalit Srimat Tirumala, Priyanka Augustine, Rebecca Anim-Boadu, Ololade Tijani, Zephton Wedderburn, Ola Rominyi, Sophie Williams.

Hull University Teaching Hospitals NHS Trust: Nathaniel Luke Hatton, Rebecca Hatton, Nur Aktar, Sanjay Dixit.

Beaumont Hospital: Suzanne Murphy, Gerard Lavelle, Mohammed Abdelsadig, Kate Connor, Yvonne Kirwan, Eloise Cowie, Riya Sharma.

The Christie NHS Foundation Trust: Marianna Theodoulou, Karan Patel.

Lancashire Teaching Hospitals NHS Foundation Trust: Christopher Chan, Luke McGurk, Benjamin Sanderson.

Queen’s Hospital: Amir Ameen, Alireza Shoakazemi, Louise Dulley.

Sussex Cancer Centre: Haddon Lathangue, Stephen David Robinson, Nektarios K Mazarakis, Cressida Lorimer, Edward Chandy, Giles Critchley.

Wellington Hospital: Michael Nichols, Andrew Parker.

University Hospital Southampton NHS Foundation Trust: Soham Bandyopadhyay, Carlo Lori, Sean Main, Jeng Ching.

Mid and South Essex NHS Foundation Trust: Femi Adeoye, George Sioftanos.

Mount Vernon Cancer Centre: Sabihya Wontumi, Swarna Arumugam, Thomas Carter.

The Northern Ireland Cancer Centre: David Conkey, Jacqui Harney.

University Hospitals Bristol NHS Foundation Trust: Lindsay Rayner, Lorna Hawley.

Waikato Hospital: Sung-Min Jun, Heta Leinonen.

The Royal Marsden Hospital NHS Foundation Trust: James de Boisanger, Babushka Kalra, Antonia Creak, Liam Welsh.

Cork University Hospital: Lena Dablouk, Chantalle Berkhout, Jack Gleeson.

Christchurch Hospital: Benjamin Harley, Simon John.

University Hospitals of Leicester NHS Trust: Anupama Vjay, Shradha Bhagani.

Royal Stoke University Hospital: Adam Fullagar, Sumera Butt.

Gloucestershire Hospitals NHS Foundation Trust: Praveen TT Durage, Samir Guglani.

University Hospitals Plymouth NHS Trust: Lucy Counsell, Ellie Eddlmann, Elizabeth Lim.

North Wales Cancer Centre: Mohammed Hatata, Heba Shebani, Iffat Jurfa, Mazin Sirelkhatim.

Portsmouth Hospitals University NHS Trust: Elizabeth Wilson, Eleni Simpson.

Norfolk and Norwich University Hospitals NHS Foundation Trust: Andrew Ho.

University Hospitals Dorset NHS Foundation Trust: Jason Bowie, Mark Noble.

University Hospitals of Derby and Burton NHS Foundation Trust: Katherine Winfield, Vishnu Pillai, Mike Smith-Howell.

Barts Health NHS Trust: Paolo de Luna, Timothy Hill, Pavlos Christodoulou, Rachel Lewis.

Royal Surrey Cancer Centre: Rachel MacArthur, May Teoh.

Imperial College Healthcare NHS Trust: Mahbuba Choudhury, Miranda Bowman, Camilla Cavilli, Matthew Williams.

NHS Grampian: Adenike Williams, Rafael Moleron.

Torbay and South Devon NHS Foundation Trust: Timothy Norris.

Velindre University NHS Trust: Jennifer Golten, Tasia Aghadiuno, Jillian MacLean, James Powell.

Royal Devon University Healthcare NHS Foundation Trust: Sarah Kingdon, Anne McCormack.

East Suffolk and North Essex NHS Foundation Trust: Natalie Wheatley, Diana Lobo, Leila Bidwell, Jennifer Collins.

James Cook Hospital: Kohgulakuhan Yogalingam, Nick Wadd.

Royal Cornwall Hospitals NHS Trust: Daniel Duffy, Grant Stewart.

NHS Tayside: Sue Een Lau, Hannah Lord.

Swansea Bay University Health Board: Jennifer Kahan.

United Lincolnshire Hospitals NHS Trust: Gbenro Olukiran, Laura Walsh, Aurora Sanz-Torres.

Royal Berkshire NHS Foundation Trust: Ed Morgan Ruth Davis.

Dunedin Hospital: Michael Heckelmann, Ahmad Taha, Giles Critchley.
