# Supplementary material for: Understanding the difference in symptoms and outcomes between glioblastoma patients diagnosed based on histological or molecular criteria: a retrospective cohort analysis from the Histo-Mol GBM collaborative
Source: J Neurooncol. 2026 Jan 8;176(2):157. doi: 10.1007/s11060-025-05364-8 (PMC12783167; doi:10.1007/s11060-025-05364-8)
Supplement: Supplementary file 2 — Supplementary Material 2 [file 11060_2025_5364_MOESM2_ESM.docx]

**Title: Understanding the difference in symptoms and outcomes between glioblastoma patients diagnosed based on histological or molecular criteria: a retrospective cohort analysis from the Histo-Mol GBM collaborative.**

**Appendix 2. Participating Centres ordered by number of patients included by country:**

**United Kingdom:**

The Beatson West of Scotland Cancer Centre, NHS Greater Glasgow and Clyde, Glasgow, United Kingdom.

University Hospitals Birmingham NHS Foundation Trust, Birmingham, United Kingdom.

Northern Centre for Cancer Care, Newcastle upon Tyne Hospitals NHS Foundation Trust, Newcastle, United Kingdom.

Leeds Cancer Centre, Leeds Teaching Hospitals NHS Trust, Leeds, United Kingdom.

Clatterbridge Cancer Centre, Wirral University Teaching Hospitals NHS Foundation Trust, Liverpool, United Kingdom.

Kent Oncology Centre, Maidstone and Tunbridge Wells NHS Trust, Maidstone, United Kingdom.

Nottingham University Hospitals NHS Trust, Nottingham, United Kingdom.

Cambridge University Hospitals NHS Foundation Trust, Cambridge, United Kingdom.

National Hospital for Neurology and Neurosurgery, University College London Hospitals NHS Foundation Trust, London, United Kingdom.

Weston Park Cancer Hospital, Sheffield Teaching Hospitals NHS Foundation Trust, Sheffield, United Kingdom.

Castle Hill Hospital, Hull University Teaching Hospitals NHS Trust, Hull, United Kingdom.

The Christie NHS Foundation Trust, Manchester, United Kingdom.

Preston Cancer Centre, Lancashire Teaching Hospitals NHS Foundation Trust, Preston, United Kingdom.

Queen’s Hospital, Barking, Havering and Redbridge University Hospitals NHS Trust, Romford, United Kingdom.

Sussex Cancer Centre, University Hospitals Sussex NHS Foundation Trust, Brighton, United Kingdom.

University Hospital Southampton NHS Foundation Trust, Southampton, United Kingdom.

Mid and South Essex NHS Foundation Trust, Southend, United Kingdom.

Mount Vernon Cancer Centre, East and North Hertfordshire NHS Trust, Northwood, United Kingdom.

The Northern Ireland Cancer Centre, Belfast Health and Social Care Trust, Belfast, United Kingdom.

University Hospitals Bristol NHS Foundation Trust, Bristol, United Kingdom.

The Royal Marsden Hospital NHS Foundation Trust, London, United Kingdom.

University Hospitals of Leicester NHS Trust, Leicester, United Kingdom.

Royal Stoke University Hospital, Stoke-on-Trent, United Kingdom.

Gloucestershire Hospitals NHS Foundation Trust, Gloucester, United Kingdom.

University Hospitals Plymouth NHS Trust, Plymouth, United Kingdom.

North Wales Cancer Centre, Betsi Cadwaladr University Health Board, Rhyl, United Kingdom.

Queen Alexandra Hospital, Portsmouth Hospitals University NHS Trust, Portsmouth, United Kingdom.

Norfolk and Norwich University Hospitals NHS Foundation Trust, Norwich, United Kingdom.

University Hospitals Dorset NHS Foundation Trust, Dorset, United Kingdom.

University Hospitals of Derby and Burton NHS Foundation Trust, Derby, United Kingdom.

Barts Health NHS Trust, London, United Kingdom.

Royal Surrey Cancer Centre, Royal Surrey NHS Foundation Trust, Guildford, United Kingdom.

Charing Cross Hospital, Imperial College Healthcare NHS Trust, London, United Kingdom.

Aberdeen Royal Infirmary, NHS Grampian, Aberdeen, United Kingdom.

Torbay and South Devon NHS Foundation Trust, Torbay, United Kingdom.

Velindre University NHS Trust, Cardiff, United Kingdom.

Royal Devon University Healthcare NHS Foundation Trust, Exeter, United Kingdom.

East Suffolk and North Essex NHS Foundation Trust, Colchester, United Kingdom.

James Cook Hospital, South Tees Hospitals NHS Foundation Trust, Middlesbrough, United Kingdom.

Royal Cornwall Hospitals NHS Trust, Truro, United Kingdom.

Ninewells Hospital, NHS Tayside, Dundee, United Kingdom.

Swansea Bay University Health Board, Swansea, United Kingdom.

United Lincolnshire Hospitals NHS Trust, Lincoln, United Kingdom.

Royal Berkshire NHS Foundation Trust, Reading, United Kingdom.

**Republic of Ireland:**

Beaumont Hospital, Dublin, Republic of Ireland.

Cork University Hospital, Cork, Republic of Ireland.

**New Zealand:**

Auckland City Hospital, Auckland, New Zealand.

Wellington Hospital, Wellington, New Zealand.

Waikato Hospital, Hamilton, New Zealand.

Christchurch Hospital, Christchurch, New Zealand.

Dunedin Hospital, Dunedin, New Zealand.
