# Supplementary material for: Understanding the difference in symptoms and outcomes between glioblastoma patients diagnosed based on histological or molecular criteria: a retrospective cohort analysis from the Histo-Mol GBM collaborative
Source: J Neurooncol. 2026 Jan 8;176(2):157. doi: 10.1007/s11060-025-05364-8 (PMC12783167; doi:10.1007/s11060-025-05364-8)

**Title: Understanding the differences between glioblastoma patients diagnosed based on histological or molecular criteria: a retrospective cohort analysis from the Histo-Mol GBM collaborative.**

**Appendix 3. Data Collection Fields:**


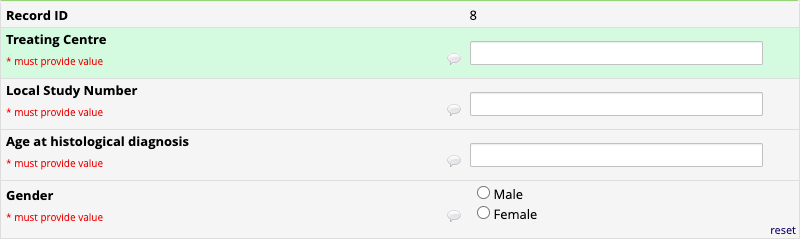

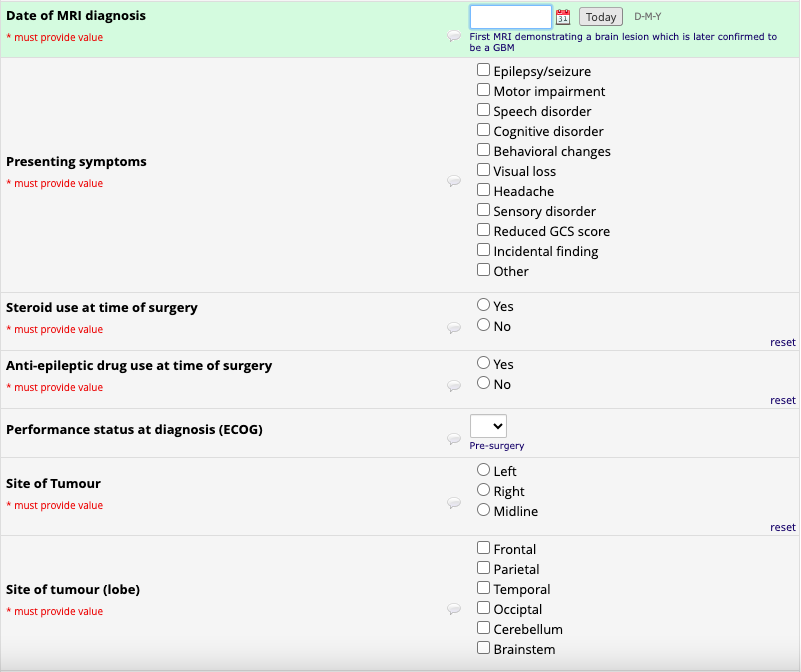

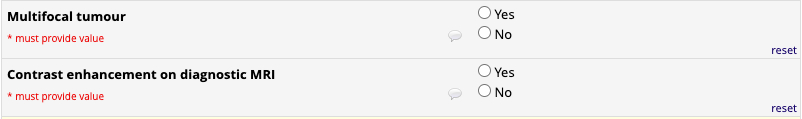

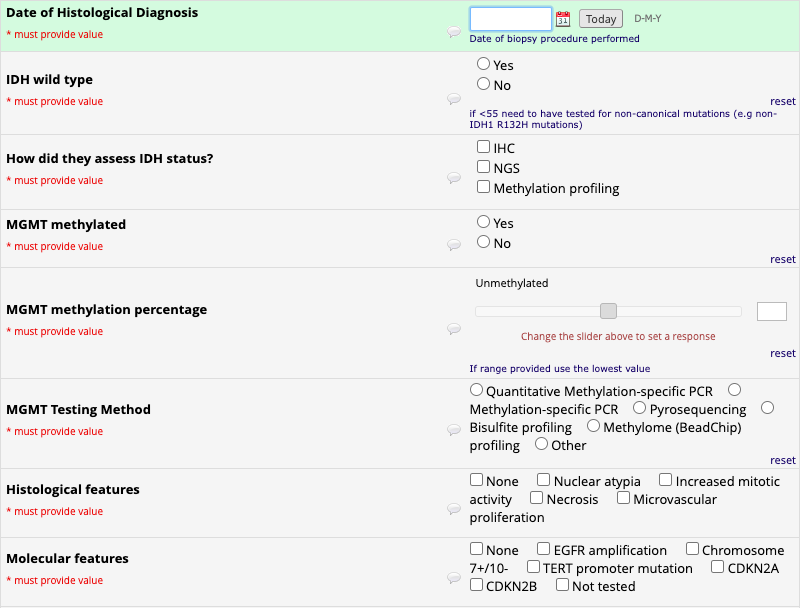

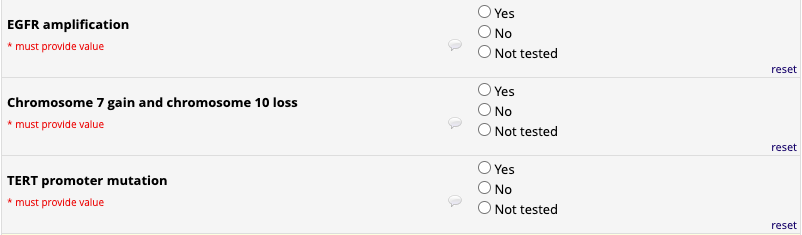

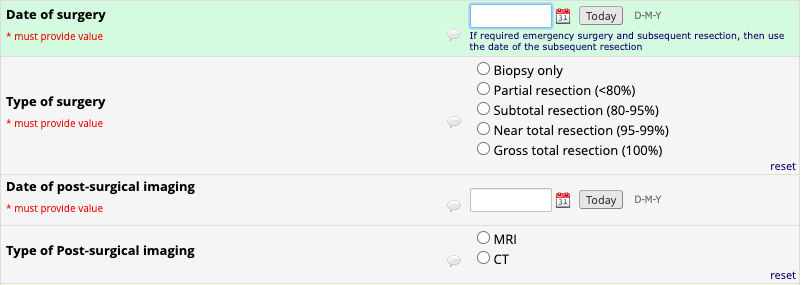

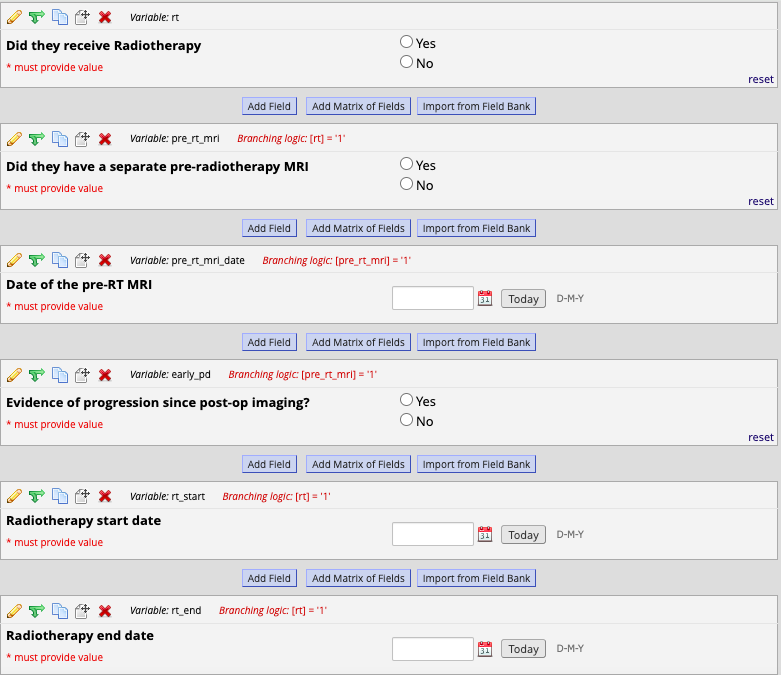

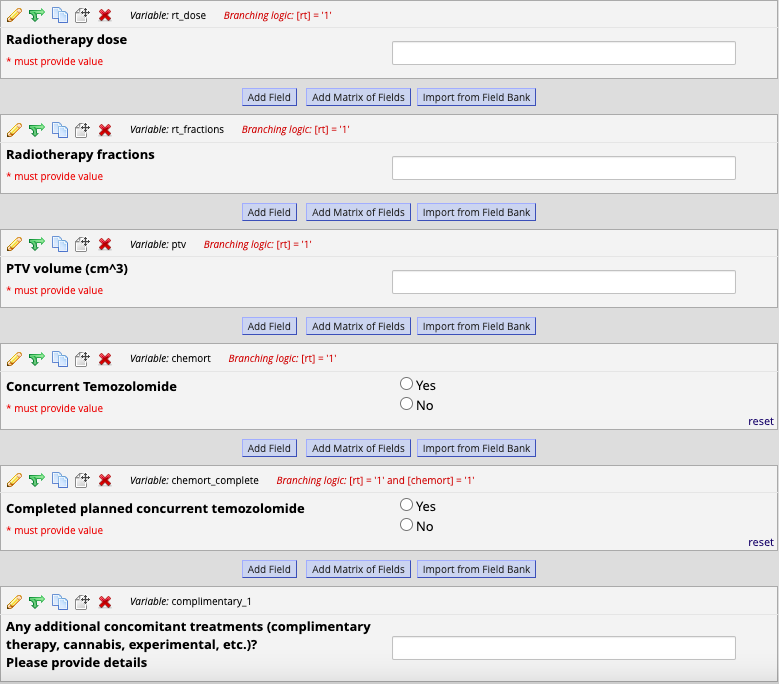

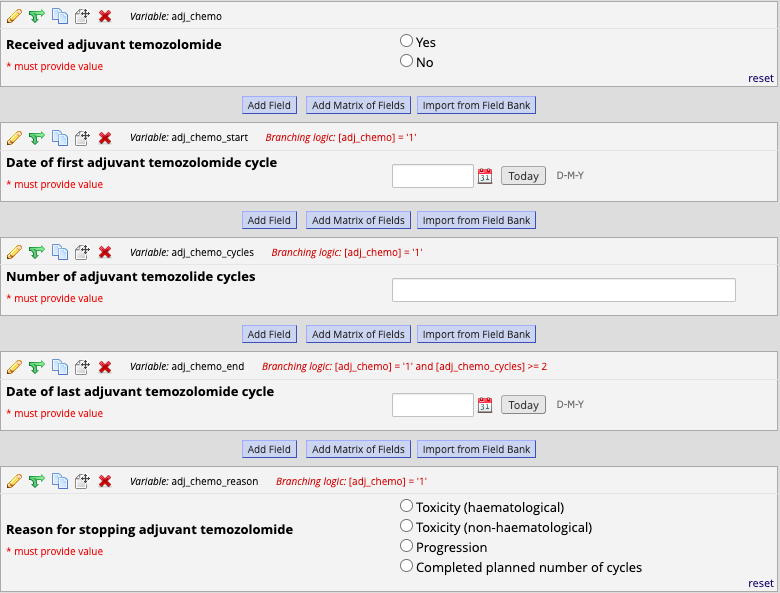

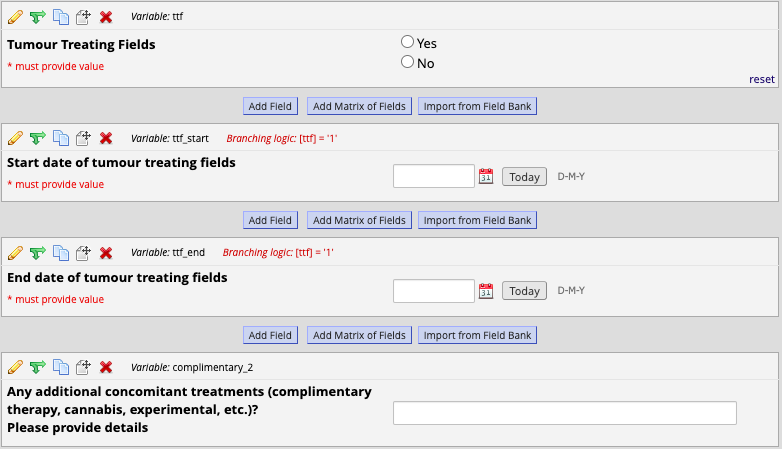

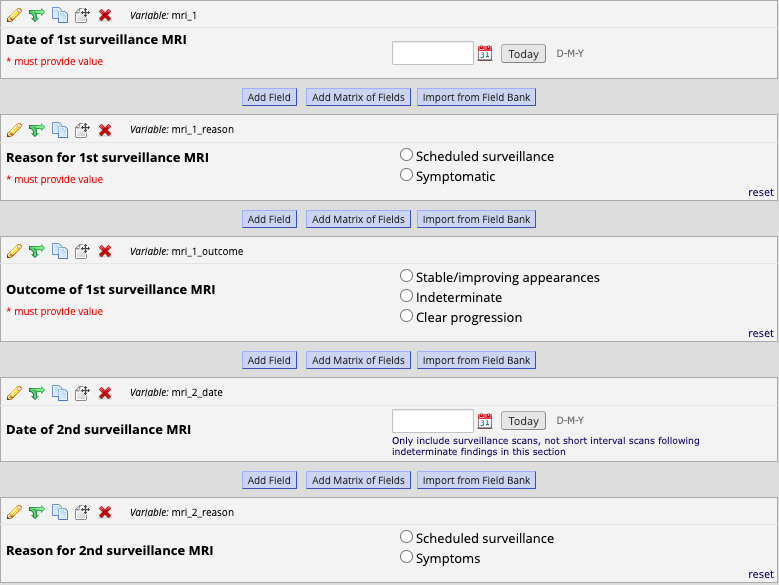

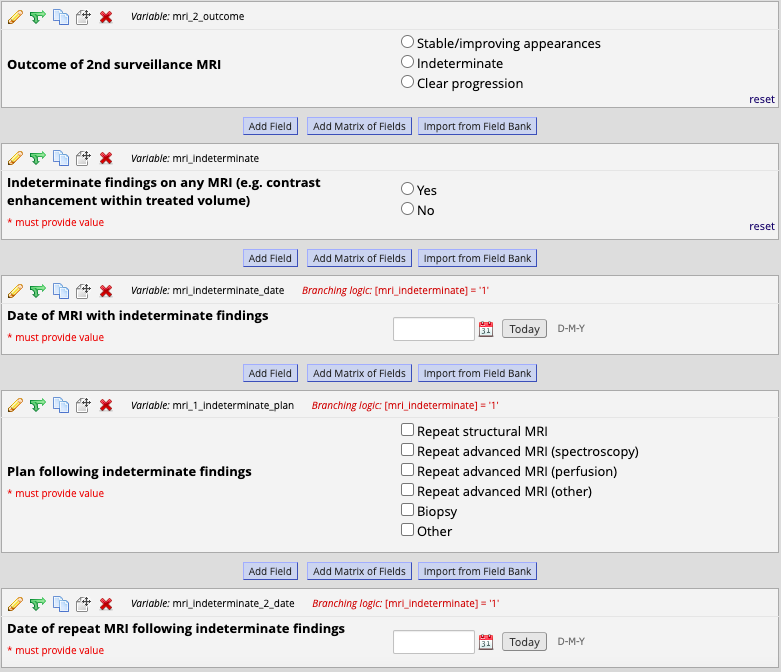

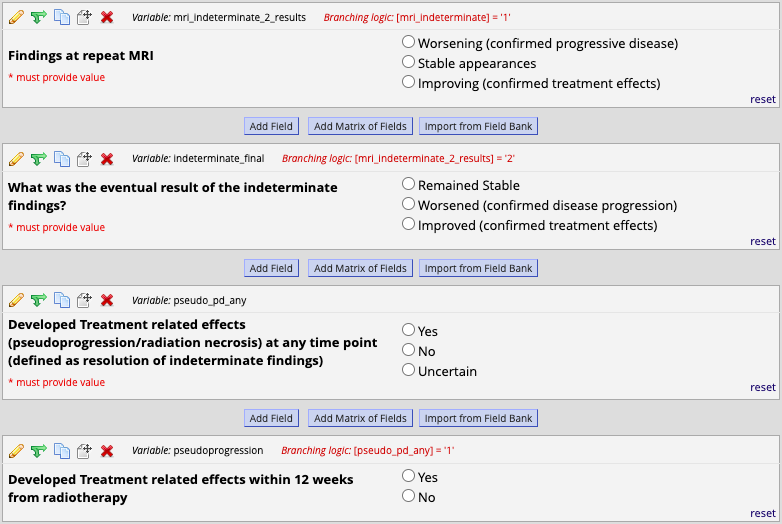

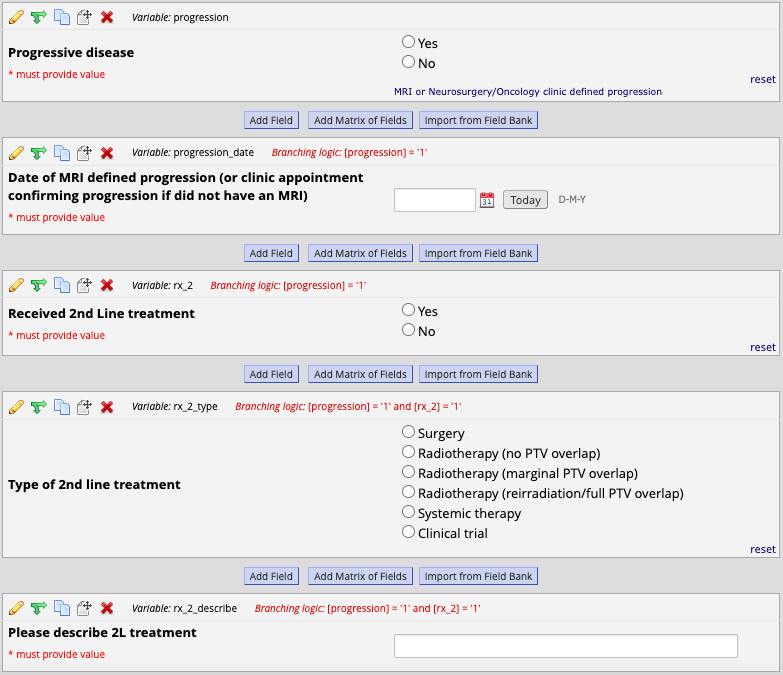

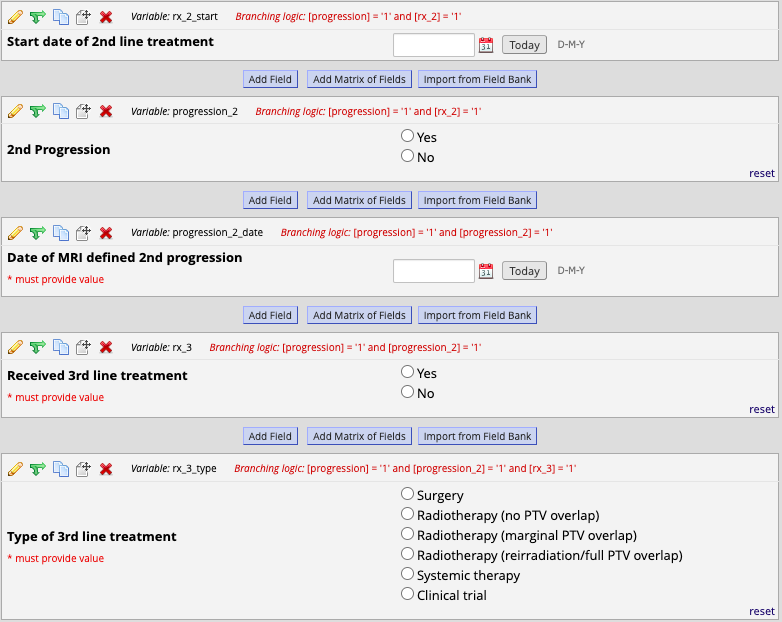

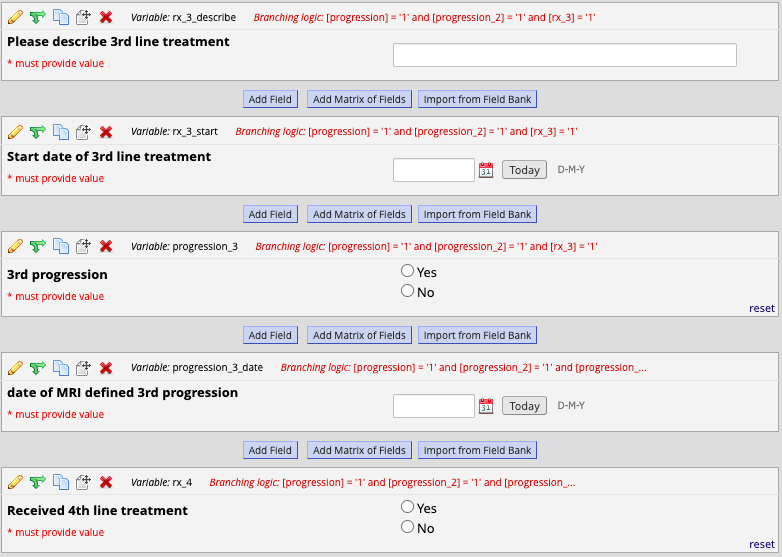

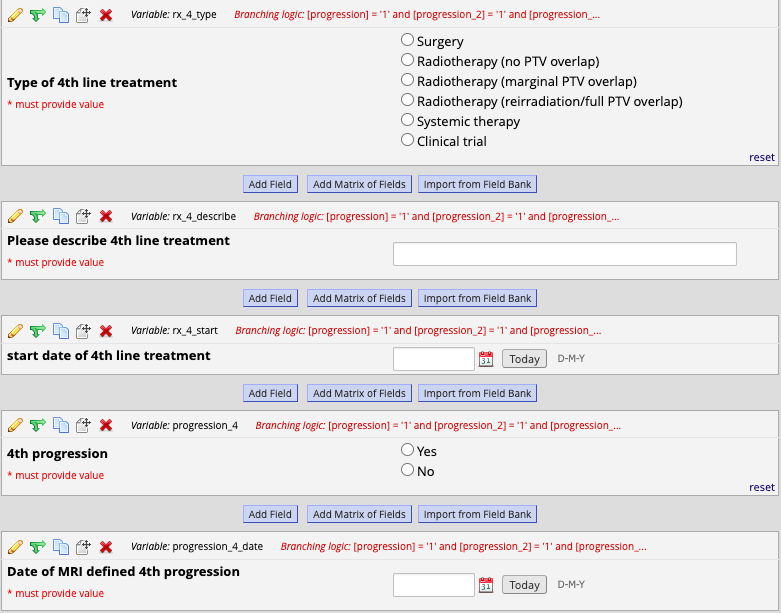

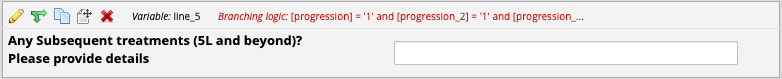

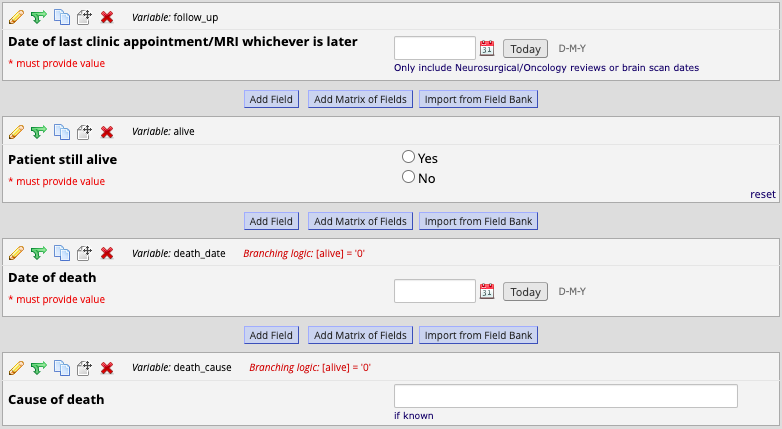

Supplement: Supplementary file 3 — Supplementary Material 3 [file 11060_2025_5364_MOESM3_ESM.docx]
