# Supplementary material for: Understanding the difference in symptoms and outcomes between glioblastoma patients diagnosed based on histological or molecular criteria: a retrospective cohort analysis from the Histo-Mol GBM collaborative
Source: J Neurooncol. 2026 Jan 8;176(2):157. doi: 10.1007/s11060-025-05364-8 (PMC12783167; doi:10.1007/s11060-025-05364-8)
Supplement: Supplementary file 4 — Supplementary Material 4 [file 11060_2025_5364_MOESM4_ESM.docx]

**Supplementary Figures:**


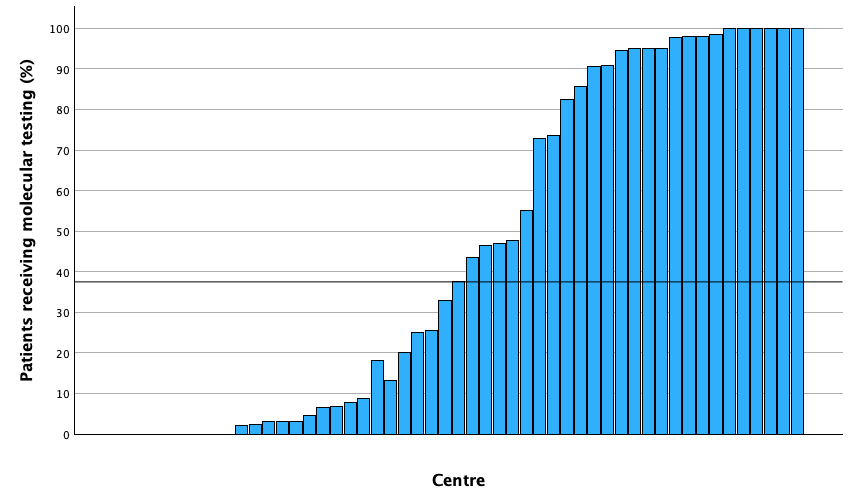


Supplementary Figure 1. Bar graph demonstrating the percentage of patients whose tumours underwent molecular testing per centre. The median percentage of patients’ tumours undergoing molecular testing (37.5%) is highlighted by the horizontal line.
