# Supplementary material for: Understanding the difference in symptoms and outcomes between glioblastoma patients diagnosed based on histological or molecular criteria: a retrospective cohort analysis from the Histo-Mol GBM collaborative
Source: J Neurooncol. 2026 Jan 8;176(2):157. doi: 10.1007/s11060-025-05364-8 (PMC12783167; doi:10.1007/s11060-025-05364-8)
Supplement: Supplementary file 5 — Supplementary Material 5 [file 11060_2025_5364_MOESM5_ESM.docx]

**Supplementary Tables:**

| Patient group | Extent of resection | Histological glioblastoma | | Molecular glioblastoma | |
| --- | --- | --- | --- | --- | --- |
|  |  | Count | % | Count | % |
| All  patients | Partial resection (<80%) | 181 | 14.9% | 5 | 21.7% |
|  | Subtotal resection (80-94%) | 406 | 33.4% | 8 | 34.8% |
|  | Near total resection (95-99%) | 289 | 23.8% | 4 | 17.4% |
|  | Gross total resection (100%) | 338 | 27.8% | 6 | 26.1% |
| Contrast  enhancing  tumours  only | Partial resection (<80%) | 175 | 15.2% | 5 | 23.8% |
|  | Subtotal resection (80-94%) | 382 | 33.1% | 7 | 33.3% |
|  | Near total resection (95-99%) | 282 | 24.5% | 4 | 19.0% |
|  | Gross total resection (100%) | 314 | 27.2% | 5 | 23.8% |

Supplementary Table 1. Exploratory analysis of extent of resection according to a modified RANO resect criteria for glioblastoma patients diagnosed according to histological criteria compared to molecular criteria.

| Variable | Binomial logistic regression |
| --- | --- |
|  | p value |
| Presenting symptom - Seizure: | 0.255 |
| **Presenting symptom - Motor:** | **0.005** |
| Presenting symptom - Sensory: | 0.081 |
| **Presenting symptom - Speech:** | **0.010** |
| Presenting symptom - Cognition: | 0.100 |
| Presenting symptom - Headache: | 0.111 |
| Presenting symptom - Incidental: | 0.255 |
| **Corticosteroids at diagnosis:** | **0.021** |
| **Anti-epileptic drugs at diagnosis:** | **0.014** |
| **Tumour Location - Parietal:** | **0.006** |
| **Tumour Location - Cerebellum** | **0.041** |
| Multifocal/multicentric tumour: | 0.423 |
| **Molecular markers – Telomerase reverse transcriptase promoter mutation:** | **<0.001** |
| **Time from MRI to surgery:** | **0.001** |
| **Extent of surgery – biopsy vs resection:** | **<0.001** |

**Supplementary Table 2. Binomial logistic regression to identify patient, tumour and treatment features associated with a diagnosis of molecular glioblastoma with the significant features highlighted in bold. MRI: magnetic resonance imaging.**
